# Localized Energy Band Bending in ZnO Nanorods Decorated with Au Nanoparticles

**DOI:** 10.3390/nano11102718

**Published:** 2021-10-14

**Authors:** Luca Bruno, Vincenzina Strano, Mario Scuderi, Giorgia Franzò, Francesco Priolo, Salvo Mirabella

**Affiliations:** 1 Department of Physics and Astronomy “Ettore Majorana”, University of Catania, Via S. Sofia 64, 95123 Catania, Italy; luca.bruno@dfa.unict.it (L.B.); stravicky@hotmail.it (V.S.); francesco.priolo@ct.infn.it (F.P.); 2 IMM-CNR, Via S. Sofia 64, 95123 Catania, Italy; giorgia.franzo@ct.infn.it; 3 IMM-CNR, VIII Strada 5, 95121 Catania, Italy; mario.scuderi@imm.cnr.it

**Keywords:** Au nanoparticles synthesis, zinc oxide nanorods, decoration, energy bands modification, luminescence, halo effect

## Abstract

Surface decoration by means of metal nanostructures is an effective way to locally modify the electronic properties of materials. The decoration of ZnO nanorods by means of Au nanoparticles was experimentally investigated and modelled in terms of energy band bending. ZnO nanorods were synthesized by chemical bath deposition. Decoration with Au nanoparticles was achieved by immersion in a colloidal solution obtained through the modified Turkevich method. The surface of ZnO nanorods was quantitatively investigated by Scanning Electron Microscopy, Transmission Electron Microscopy and Rutherford Backscattering Spectrometry. The Photoluminescence and Cathodoluminescence of bare and decorated ZnO nanorods were investigated, as well as the band bending through Mott–Schottky electrochemical analyses. Decoration with Au nanoparticles induced a 10 times reduction in free electrons below the surface of ZnO, together with a decrease in UV luminescence and an increase in visible-UV intensity ratio. The effect of decoration was modelled with a nano-Schottky junction at ZnO surface below the Au nanoparticle with a Multiphysics approach. An extensive electric field with a specific halo effect formed beneath the metal–semiconductor interface. ZnO nanorod decoration with Au nanoparticles was shown to be a versatile method to tailor the electronic properties at the semiconductor surface.

## 1. Introduction

Zinc oxide (ZnO) is an *n*-type semiconductor (band gap of 3.2–3.4 eV, large excitonic-binding energy of 60 meV) attracting great attention due to its physical and chemical properties [1,2]. In particular, ZnO nanorods (NRs) have proven to be promising nanostructures for a wide range of applications, especially for photonics and optoelectronics in the UV or blue spectral range [3,4,5,6,7,8,9]. A controlled improvement of performance needs a microscopic understanding of ZnO surface states and deep levels, especially in low-dimensional nanostructures where the significant surface-to-bulk ratio significantly impacts electronic energy band bending.

The surface decoration of semiconductor nanostructures with metallic nanoparticles (NPs) usually leads to an improvement of their catalytic and electrical properties [10,11,12,13,14]. The formation of nano-Schottky junctions at the metal–semiconductor interface leads to the creation of a strong electric field directed toward the surface and to a significant modification of the ZnO NRs energy band profiles [15,16,17,18,19]. As a consequence, space charge regions and surface-localized electric fields promote a catalytic effect and modify the radiative recombination process. Apart from near-band-edge emission, ZnO nanostructures may also exhibit luminescence in the visible range [1,20,21,22,23,24,25,26,27]. When decorated, luminescence from ZnO NRs changes, with an enhancement of visible emission at the expense of UV emission [10,19,28,29]. Such a process could be the consequence of a significant bending of electronic energy bands into ZnO just below the metal nanoparticle, inducing free carrier depletion. Such an effect in decorated ZnO nanorods allows the application of these composite materials in UV sensing and light-induced catalysis [11,16,18,19,30,31].

In this work, we systematically investigated the decoration of ZnO NRs with Au NPs and its effect on position and population of electronic energy bands. Photoluminescence and cathodoluminescence were used to exploit the decoration effect to different extents. Energy band modifications and carrier concentrations were also investigated through electrochemical analyses and simulated using a multiphysics approach, revealing a noticeable halo effect in the electric field at the ZnO surface close to the edges of the Au NPs.

## 2. Materials and Methods

### 2.1. Synthesis and Decoration of ZnO Nanorods

ZnO NRs were synthesized through chemical bath deposition (CBD) on Si substrates cut by Czochzralski (Cz) wafers. Zinc nitrate hexahydrate (Zn(NO_3_)_2_·6H_2_O, Sigma-Aldrich, St. Louis, MO, USA, ≥99%) and hexamethylenetetramine (HMTA, Sigma-Aldrich, St. Louis, MO, USA, ≥99.5%) were used for the growth of ZnO NRs with concentrations of 25 mM and 50 mM, respectively [32].

Au NPs were synthesized through the modified Turkevich method [33,34,35] at room temperature, without any correction of the pH of the solution (see Supplementary Information). Gold chloride trihydrate (HAuCl_4_·3H_2_O, Sigma-Aldrich, St. Louis, MO, USA, ≥99.9%) and trisodium citrate (Na_3_C_6_H_5_O_7_·H_2_O, Sigma-Aldrich, St. Louis, MO, USA) were used without further purification.

ZnO NRs were decorated via dip coating by simply immersing different substrates in the Au NP colloidal solution. Decorated samples are labelled according to the number of immersions in the solution (e.g., Au_5_-ZnO refers to a ZnO NR immersed 5 times into Au NP solution).

### 2.2. Characterization

UV-vis spectroscopy was performed on the Au solution using a Varian Cary 500 (Agilent Technologies, Santa Clara, CA, USA) double beam scanning UV/VIS/NIR spectrophotometer.

Surface morphology was analyzed by using a Scanning Electron Microscope (Gemini field emission SEM Carl Zeiss SUPRA 25, FEG-SEM, Carl Zeiss Microscopy GmbH, Jena, Germany). SEM images were analyzed using ImageJ software [36].

Transmission electron microscopy (TEM) analyses of Au NPs dispersed on a TEM grid were performed with a Cs-probe-corrected JEOL JEM ARM200F microscope at a primary beam energy of 200 keV operated in scanning TEM (STEM) mode.

The amount of Au loading onto ZnO NRs was evaluated by Rutherford backscattering spectrometry (RBS, 2.0 MeV ${\mathrm{He}}^{+}$ beam at normal incidence) with a 165° backscattering angle using a 3.5 MV HVEE Singletron accelerator system (High Voltage Engineering Europa, The Netherlands). RBS spectra were analyzed using XRump software [37].

Photoluminescence (PL) measurements were performed by pumping at ∼0.7 mW the 325 nm (3.81 eV) line of a He–Cd laser chopped through an acousto-optic modulator at a frequency of 55 Hz. The PL signal was analyzed using a single grating monochromator, detected with a Hamamatsu visible photomultiplier, and recorded with a lock-in amplifier using the acousto-optic modulator frequency as a reference. PL spectra were taken in air or vacuum within a cryostat ($∼10^{6}$ mbar) to ascertain the role of atmospheric O_2_. All PL spectra were converted by Jacobian transformation from wavelength to energy dispersion [38].

Cathodoluminescence (CL) measurements were performed using a Scanning Electron Microscope (Gemini field emission SEM Carl Zeiss SUPRA 25, FEG-SEM, Carl Zeiss Microscopy GmbH, Jena, Germany), equipped with a Gatan MonoCL3 CL spectroscopy/imaging system. The beam energy was varied between 2 and 20 keV, providing different probe depths below the surface as determined by *CASINO* simulations [39]. All CL spectra were corrected for the overall detection response of the system.

Electrochemical measurements were carried out at room temperature by using a VersaSTAT 4 potentiostat (Princeton Applied Research, Oak Ridge, TN, USA) and a three-electrode setup with a platinum counter electrode, a saturated calomel electrode (SCE) as reference electrode, and the samples as working electrodes. An amount of 0.5 M $Na_{2}SO_{4}$ (Sigma Aldrich, St. Louis, MO, USA, ≥85%) was used as a supporting electrolyte. Mott–Schottky (M-S) analyses were conducted on bare and decorated ZnO NRs samples in the potential range −1÷0 V vs. SCE, at 1000 Hz frequency.

A simulation of the band position of the semiconductor and the electric field on the surface of ZnO NRs induced by the metal decoration has been carried out by *COMSOL Multiphysics*^®^ *software* (v.5.0, COMSOL Inc., Stockholm, Sweden) [40] (details in Supplementary Information).

## 3. Results and Discussion

**Figure 1:**
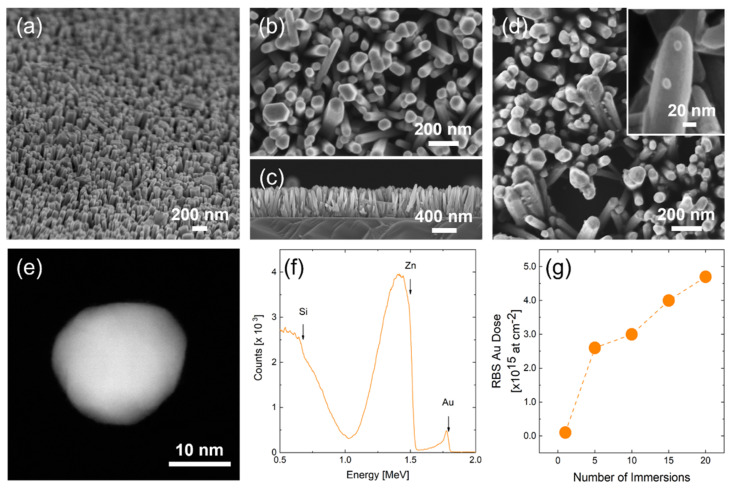
(**a**) Tilted SEM images of bare ZnO NRs; (**b**) magnified SEM image of ZnO NRs and (**c**) cross section SEM image of ZnO nanostructured film; (**d**) SEM images at different magnifications of Au_20_-ZnO; (**e**) STEM image of a Au NP; (**f**) RBS spectrum of the Au_20_-ZnO sample; (**g**) variation of the Au loading on the surface of ZnO NRs as a function of the number of immersions.

The CBD grown ZnO nanorods are shown in Figure 1a–c. A uniform nanostructured film made of ZnO NRs grown onto Si can be seen in low magnification SEM image in Figure 1a. ZnO NRs present a width of approximately 100 nm and a length of 700 nm (Figure 1b,c). To decorate these ZnO NRs, we immersed this sample into the Au colloidal solution. The presence of Au nanoparticles within the colloidal solution produced with the modified Turkevich method was confirmed with UV-vis spectroscopy over two months (Figure S1). Both spectra showed a narrow and sharp peak (centered at around 530 nm) which was a clear indication of the presence of stable NPs in colloidal form [35].

The effective decoration of ZnO NRs with metal NPs can be appreciated in Figure 1d, showing ZnO NRs with the surface densely decorated by Au NPs after 20 immersions (inset of Figure 1d). The NPs showed a reasonably uniform diameter of about 20±3 nm, as measured by SEM images on more than 200 NPs dispersed on a flat Si substrate. A STEM picture of a representative Au nanoparticle is shown in Figure 1e, confirming the 20 nm size. Each nanoparticle has a rounded shape with some bumps due to the crystalline grains of which it is comprised (see Supplementary Information, Figure S2). RBS analyses (Figure 1f) confirmed the presence of a small amount of Au (peak at 1.8 MeV) and Zn (large peak at 1.0–1.5 MeV) onto Si (signal from 0.6 MeV downward), as expected. RBS was used to perform a quantitative measurement of Au after multiple immersion, since the Au amount is proportional to the Au peak in the spectrum [19]. Figure 1g shows the variation in Au amounts with the number of immersions, showing a fairly linear increase from $1.0\times 10^{14}\;{\mathrm{at}\;\mathrm{cm}}^{-2}$ (after 1 immersion) to $4.7\times 10^{15}\;{\mathrm{at}\;\mathrm{cm}}^{-2}$ (after 20 immersions). As expected, Au amounts on ZnO NRs increased with the number of immersions, allowing carefully monitoring of the extent of decoration. We verified that the size of Au NPs does not change with the number of immersions, confirming that the synthesis method produces highly stable suspension of Au NPs [33,35]. Our decoration method effectively covered the ZnO NRs surface with a varying density of Au NPs.

Au NP density cannot be ascertained by SEM analysis because of the rough surface and shadowing effect. Thus, the Au amount ($D_{RBS}$, obtained by RBS [41]) was joined with Au NP diameter to evaluate the density N of NPs decorating ZnO NRs, through the following relation:

(1) $$D_{RBS}=N\;\rho_{at}\;V_{NP}$$

where $\rho_{at}$ is the Au atomic bulk density $\left(5.9\times 10^{22}{\;\mathrm{at}\;\mathrm{cm}}^{-3}\right)$ and $V_{NP}$ is the volume of a single NP (${\mathrm{cm}}^{3}$) based on the measured size of NPs. The result of such an exercise shows that the NP density can be tuned from $1.0\times 10^{9}\;{\mathrm{NPs}\;\mathrm{cm}}^{-2}$ to $4.5\times 10^{10}{\;\mathrm{NPs}\;\mathrm{cm}}^{-2}$ (from 1 up to 20 immersions).

**Figure 2:**
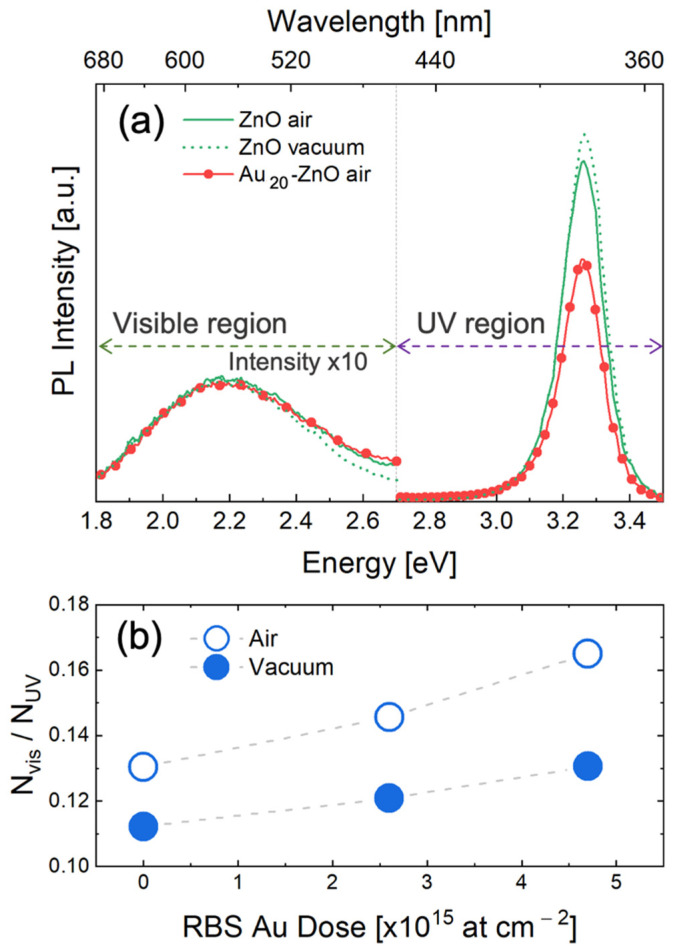
(**a**) PL spectra of bare ZnO (green continuous line) and Au_20_-ZnO (red and dotter line) in air, and bare ZnO in vacuum (green dotted line), with visible emission multiplied by a factor of 10; (**b**) ratio between the intensity of visible ($N_{vis}$) and UV emission ($N_{UV}$) in air and in vacuum versus Au NP-decoration extent.

PL spectra were acquired for both bare and decorated NRs in vacuum and in air (Figure 2). All the emission spectra (Figure 2a) consisted of a UV region (2.7–3.5 eV) and a visible region (1.8–2.7 eV). Figure 2 reports the visible emission multiplied by a factor of 10 with respect to the UV emission. It is now well-documented that a UV peak arises from a very fast transition (timescale below 1 ns [42,43,44,45,46,47,48,49]) of free excitons from a donor state (FX-D) to valence band maximum (VBM) [50]. The visible emission is due to recombination between holes in VBM and electrons trapped at midgap levels induced by oxygen vacancy ($V_{O}$) or zinc vacancy ($V_{Zn}$) [20,50,51,52,53]. This process is much slower than that leading to UV emission, with characteristic times in the ns–μs range [42,44,47,49].

PL measurement in vacuum is aimed at disentangling the effect of atmospheric $O_{2}$, as it is well-known that oxygen adsorption onto ZnO NRs modifies the energy bands at surface [51]. The visible PL is barely affected by gold decoration or $O_{2}$ adsorption, but the UV radiative recombination of electron-holes in ZnO is clearly modified. The area of each peak in the PL spectra was related to the number of photons emitted in that energy range, so the ratio ($N_{vis}/N_{UV}$) between the areas of visible and UV peaks quantified how many photons were emitted in the visible range per each photon emitted in the UV range. Such a ratio indicates the relative probability of radiative recombination in the two channels, and its changes with surface condition are indicative of modification in the electron-hole recombination process. Figure 2b displays $N_{vis}/N_{UV}$ increasing with Au NP loading, both in air and in vacuum. In addition, this ratio, as measured in air, was always higher than in vacuum. It is worth noting that Au decoration in vacuum obtained the same effect ($N_{vis}/N_{UV}$ increase from 0.11 to 0.13) as $O_{2}$ adsorption (without metal NPs). The largest visible-to-UV emission was obtained by joining the two effects on the ZnO surface ($O_{2}$ adsorption and Au decoration). This imbalance between UV and visible emission is later explained in terms of energy-band bending caused by nano-Schottky junction at the metal–semiconductor interface.

The experimental results reveal that Au NP decoration and $O_{2}$ surface adsorption show a similar effect on the radiative recombination, as they: (i) reduce the UV emission and slightly increase the visible emission; (ii) produce a high energy tail in the visible emission spectrum.

**Figure 3:**
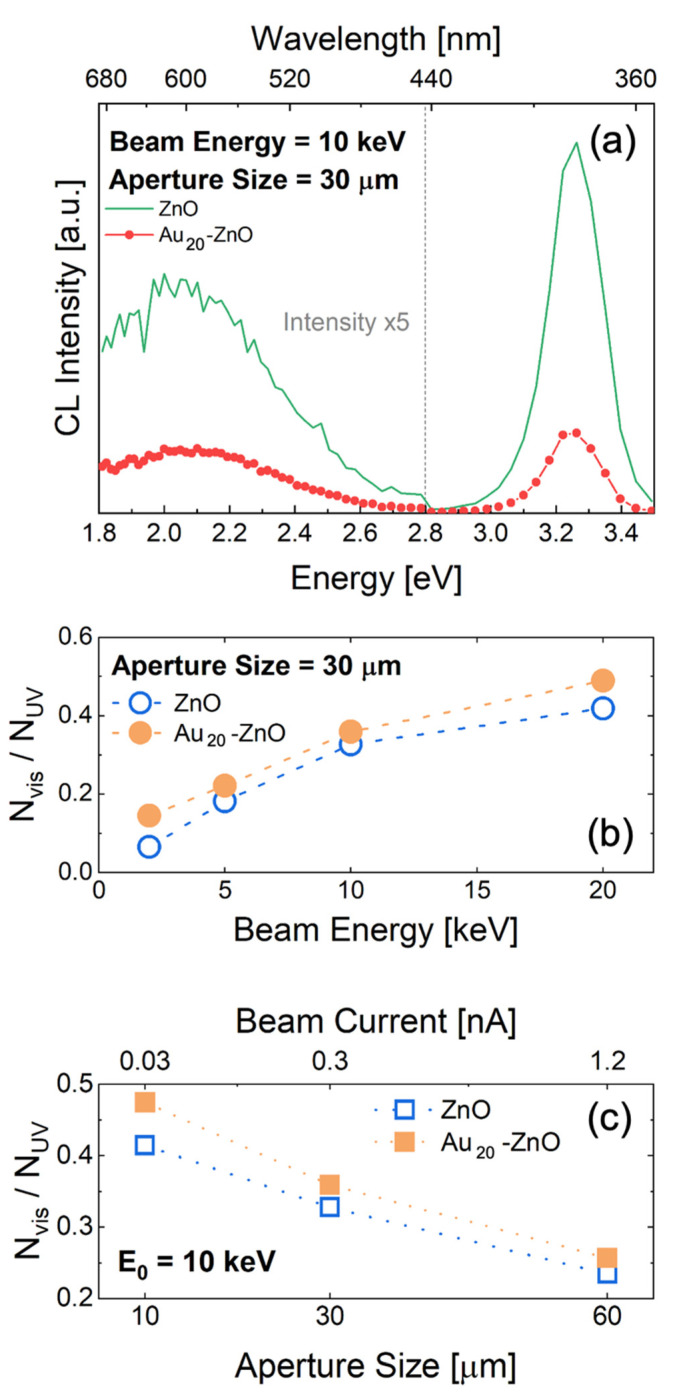
(**a**) CL spectra of bare ZnO (green) and Au_20_-ZnO (red), with visible emission multiplied by a factor of 5; ratio between the intensity of visible ($N_{vis}$) and UV emission ($N_{UV}$) as a function of the electron beam energy (**b**) and current (**c**).

To further investigate the effect of ZnO NRs decoration with Au NPs, CL analyses were carried out on bare (ZnO) and decorated samples (Au_20_-ZnO). CL spectra were acquired at different electron beam energies ($E_{0}$, varied in the range 2–20 keV) and different beam currents (using SEM aperture of 10, 30 and 60 μm). CL spectra for bare ZnO and Au_20_-ZnO at a beam energy of 10 keV are reported in Figure 3a. The CL spectra showed the emission from two different peaks, a sharp UV peak and a broad visible region (as in PL analysis). For Au_20_-ZnO (red line in Figure 3), the absolute intensity of the two peaks significantly decreased compared to that of bare ZnO, most likely because of the shadowing effect of the Au NPs in the decorated sample.

CL spectra of bare ZnO acquired for different beam energies and currents are reported in the Supplementary Information (Figure S3). Figure 3b,c show that the $N_{vis}/N_{UV}$ ratio increased with beam energy and decreased with beam current. In PL as well as CL the presence of Au NPs gave a larger $N_{vis}/N_{UV}$ ratio with respect to bare ZnO. As the beam energy increased, the probe depth also increased. The probe depths at different energies were simulated with Monte Carlo simulations through CASINO software [39,54,55,56] (Figure S4), ranging from a few tens of nanometers (at 2 keV) to 2 micrometers (at 20 keV). As a direct consequence, the generation of electron-hole pairs was diluted in a much larger volume, inducing a dramatic reduction of volumetric generation rate (Figure S4) by four orders of magnitude. This datum highlights that the $N_{vis}/N_{UV}$ ratio increased at lower e–h pair generation rates. Figure 3c clearly confirms this trend, showing variation in the beam current without a change in probe depth. These results can be explained by considering the different timescales of the two radiative recombination processes. The slow process (leading to visible emission) became weaker at a high generation rate, most likely because the strong concentration gradient of electron-hole (e–h) pairs induced a significant diffusion which reduced the recombination on the long timescales.

**Figure 4:**
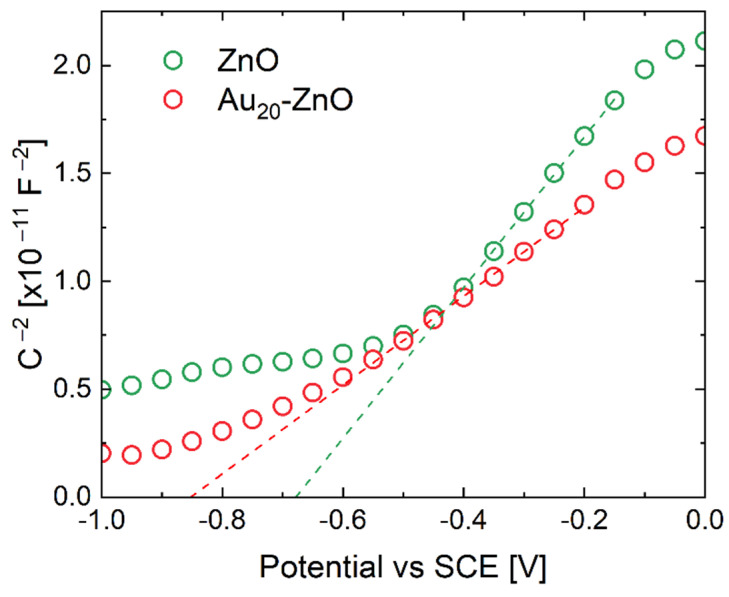
Mott–Schottky plot of ZnO and Au_20_-ZnO in 0.5 M ${\mathrm{Na}}_{2}{\mathrm{SO}}_{4}$ (dashed lines represent the fits to linear regions).

In order to better understand the effect of Au decoration on energy bands and free carrier density, Mott–Schottky analysis was performed by immersing the bare and decorated ZnO NR sample in an aqueous solution of ${\mathrm{Na}}_{2}{\mathrm{SO}}_{4}$. Even if a liquid–semiconductor interface is now tested, we wish to extract information on the Au NPs decoration effects. ZnO and Au_20_-ZnO samples were characterized by measuring their capacitance as a function of electrode potential [57,58,59,60,61,62], and a typical result is shown in the Mott–Schottky plot ($C^{-2}\;vs.\;E$) in Figure 4. The flat band potential $E_{FB}$ and the donor density $N_{D}$ can be obtained from the linear part of the plot as intercept with *x*-axis and slope, respectively (see Supplementary Information for details) [57,58,59,60,62,63,64,65]. The plot appears to have a linear section in the potential range −0.2÷−0.45 V vs. SCE in the case of ZnO, and −0.2÷−0.6 V vs. SCE for Au_20_-ZnO sample. The flat band potentials obtained were −0.68 V and −0.85 V vs. SCE for ZnO and Au_20_-ZnO, respectively. Concerning donor concentration, from Equation (S5), we found values of $3.2\times 10^{17}\;{\mathrm{cm}}^{-3}$ and $2.0\times 10^{16}\;{\mathrm{cm}}^{-3}$ for bare and decorated samples, respectively. The flat band potential gave an indication of the band bending at the liquid–semiconductor interface at equilibrium, and here we observe that such a bending was almost 0.2 eV greater in Au_20_-ZnO. This was most likely due to the Au NP decoration. On the other hand, the reduction by one order of magnitude in the donor concentration in Au_20_-ZnO confirmed that Au NP decoration effectively depleted the free carriers on the surfaces of the ZnO NRs.

## 4. Modeling the Au NP Decoration Effect

Au NP-decoration clearly affects the electronic energy band at ZnO, as largely shown in the previous section. We now attempt to quantify such an effect, investigating the band bending at the metal–semiconductor interface through a multiphysics approach.

**Figure 5:**
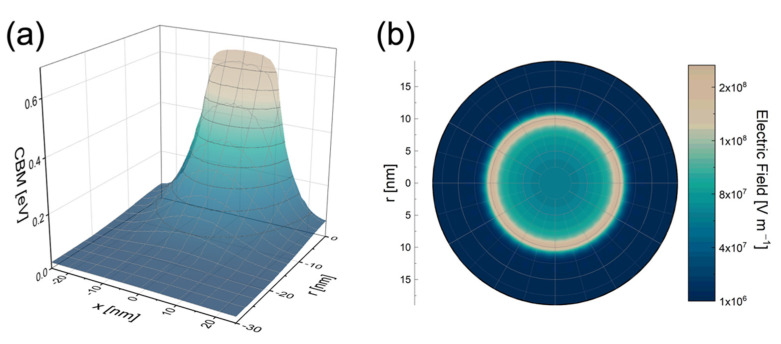
*COMSOL* simulations at the interface between Au and ZnO NR. (**a**) 3D plot showing CBM energy and (**b**) electric field map in ZnO 5 nm below a circular Au dot.

To visualize the band modification in ZnO NRs decorated with metal NPs, *COMSOL* [40] simulations were performed assuming a single Au circular dot (20 nm in size) placed onto ZnO in a vacuum ambient (see Figure S5 for a scheme of simulation). Band bending occurs at the metal–semiconductor junction because of differing work functions. Au has a higher work function ($\Phi_{\mathrm{Au}}=4.8\;\mathrm{eV}$ [66]) than that of ZnO ($\Phi_{B}=4.2\;\mathrm{eV}$ [67]), leading to a potential barrier for electrons and to a significant upwards bending of ZnO conduction and valence bands at the metal–semiconductor interface. The energy map of the conduction band minimum (CBM) as a function of depth and distance from the NP center is reported in Figure 5a. The simulation disregarded any surface defects or temperature dependence. The upward bending of the CBM surmounted 0.6 eV beneath the Au NP center and extended almost 20 nm within the ZnO material. As a consequence, electron population in the conduction band was significantly affected. Moreover, an extensive electric field arise under the Au NP, pointing Au. The 2D map of the electric field at the ZnO surface below a circular Au dot is shown in Figure 5b, showing intensity as high as $10^{8}{\;V\;m}^{-1}$ with a characteristic halo effect beneath the Au NP circumference. The electric field is proportional to the spatial derivative of the CBM energy; thus, the highest electric field is found close to the Au NP edges, creating this distinctive halo. Such a strong and localized electric field caused by Au decoration is extremely effective in modifying the band profile and carrier density close to the surface. Moreover, the halo effect may be responsible for the catalytic effect at ZnO surface sites close to Au NP edges.

To understand the PL and CL data of ZnO NRs decorated with Au NPs, the radiative mechanism in ZnO NRs must be detailed. As already stated, UV emission is a rapid process occurring a fraction of a ns after e-h generation, while visible emission at mid-gap levels is a radiative recombination of e-h much slower than that causing UV emission [42]. It has already been demonstrated that these levels are below the Fermi level [24,68,69]. Thus, UV emission is expected to be affected by band bending to a far greater degree than visible emission, as the former is related to shallow levels whose population largely depends on Fermi level position [19]. The presence of a large electric field caused by Au NP decoration (Figure 5b) could very effectively promote separation of generated charge carriers, thus reducing the rapid e-h recombination. This separation could explain the increase of $N_{vis}/N_{UV}$ ratio observed in the decorated samples (both in PL and in CL analyses).

## 5. Conclusions

In conclusion, this study reports the investigation of a simple procedure for ZnO NRs decoration with Au NPs, in which the effects on the position and population of electronic energy bands of ZnO were discussed and modelled. Surface decoration with 20 nm Au NPs was achieved by multiple immersion in a colloidal solution, leading to NP density up to $4.5\times 10^{10}\;{\mathrm{NPs}\;\mathrm{cm}}^{-2}$. Au decoration significantly affected the radiative emission of ZnO to different extents in the UV and visible emission processes. The Au decoration significantly reduced the UV radiative emission in comparison to the visible emission. Such evidence is attributed to a noteworthy upwards band bending caused by the nano-Schottky junction formed at the Au–ZnO interface. A strong electric field (up to $10^{8}{\;V\;m}^{-1}$) at ZnO surface results from multiphysics simulation, with a distinctive halo effect beneath the Au NP edges. The effect in photo- and cathodoluminescence analyses is discussed in terms of enhanced separation of generated e-h pairs.

## Data Availability

The data presented in this study are available on request from the corresponding author.

## Supplementary Materials

The following are available online at https://www.mdpi.com/article/10.3390/nano11102718/s1, Figure S1: Absorbance spectrum of Au NP solution as prepared and after 2 months; Figure S2: STEM micrograph and high magnification insets of an isolated Au NP; Figure S3: CL spectra of bare ZnO at different electron beam energies; Figure S4: (a) *CASINO* simulation of the probe depth of the electron beam at different beam energies; (b) e–h generation rate (calculated from Equation (S3)); Figure S5: 2D *COMSOL* simulation of the electric field of a single ZnO NR in presence of a Au NP on its surface.
