# Supplementary material for: Localized Energy Band Bending in ZnO Nanorods Decorated with Au Nanoparticles
Source: Nanomaterials (Basel). 2021 Oct 14;11(10):2718. doi: 10.3390/nano11102718 (PMC8539582; doi:10.3390/nano11102718)
Supplement: Supplementary file 1 [file nanomaterials-11-02718-s001.zip › nanomaterials-1391193-supplementary.pdf]

Localized energy band bending in ZnO nanorods decorated with Au nanoparticles

Luca Bruno [1,2], Vincenzina Strano [1,2], Mario Scuderi [3], Giorgia Franzò [2], Francesco Priolo [1,2] and Salvo Mirabella [1,2,*]

[1] Department of Physics and Astronomy "Ettore Majorana", University of Catania, via S. Sofia 64, 95123 Catania, Italy; luca.bruno@dfa.unict.it (L.B.); stravicky@hotmail.it (V.S.); francesco.priolo@ct.infn.it (F.P.)
[2] IMM-CNR, via S. Sofia 64, 95123 Catania, Italy; giorgia.franzo@ct.infn.it
[3] IMM-CNR, VIII Strada 5, 95121 Catania, Italy; mario.scuderi@imm.cnr.it
* Correspondence: salvo.mirabella@dfa.unict.it

Synthesis of Au nanoparticles and ZnO decoration

Au nanoparticles were synthesized at room temperature without any correction of the pH of the solution. The same volumes of 3 mM solution of trisodium citrate and 1 mM solution of gold trichloride were mixed to get the desired molar concentration ratio of 1:3, with a final volume of 50 ml. The solution was stirred up to 24 hours at room temperature in a becker covered with Parafilm (Bemis Company Inc, Zürich, Switzerland). The solution colour shows a transient colour change during the stirring and becomes stable in 2-3 hours with the characteristic red wine colour.

After the immersion, the samples were dried on a hot plate and the entire procedure was repeated up to 20 times in order to vary the amount of Au NPs on the surface of the NRs. The samples were then rinsed with deionized water (MilliQ, 18 MΩ cm) and dried in N_2 gas flow.

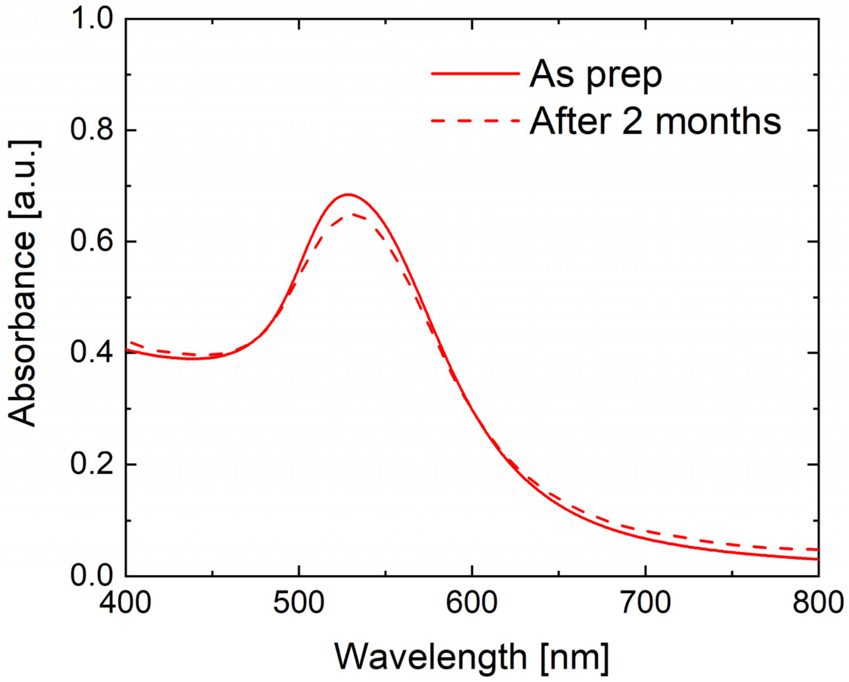

Figure S1. Absorbance spectrum of Au NPs solution as-prepared and after 2 months.

Au NPs morphology

STEM images like those in Figure S5 showed that these AuNPs exhibit highly polycrystalline structures with grain size down to 10 nm. Indeed, the higher magnification insets of Figure S5 show different crystallographic orientations arising from different grains composing the NP.

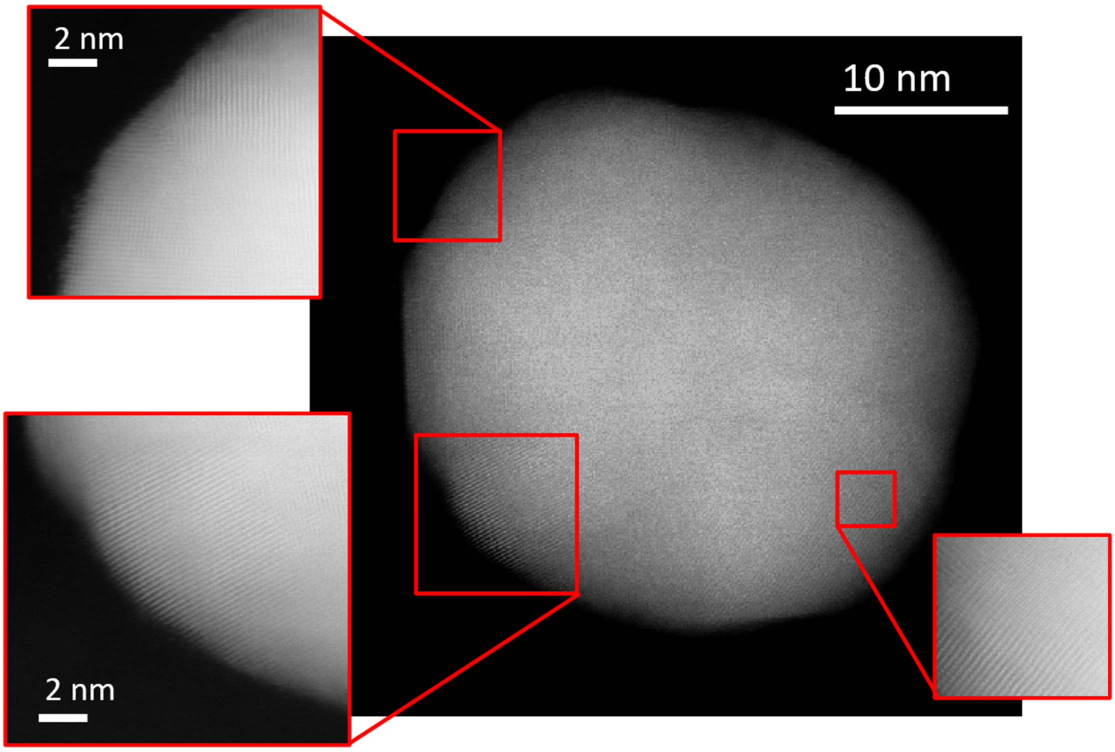

Figure S2. STEM micrograph and high magnification insets of an isolated Au NP.

Cathodoluminescence measurements

The relationship between E_0 and the probe depth R is

$$R\ [\mathrm{nm}] = \frac{27.6\ A\ E_0^{1.67}}{\rho\ Z^{0.89}} \qquad (S1)$$

where ρ is the target material density (g/cm³); Z and A are, respectively, the target atomic number and atomic weight; and E_0 (keV) is the initial electron energy.

Information from CL at different energies can be calculated as a function of Kanaya–Okayama electron penetration depth [1] using Monte Carlo simulations of electron-solid interaction. As expected, when the beam energy is increased from 2 to 20 keV, the penetration depth (and so the probe depth) passes from few nm to around 2 μm [1, 2].

As the energy of the beam varies, it is possible to calculate the e-h generation rate (G_0) from the relationship [3]

$$G_0 = 6.25\ x\ 10^{21}\ E_0\ I_0\ \frac{1 - \eta\ \bar{E}/E_0}{E_i} \qquad (S2)$$

where E_0 is the electron beam energy, I_0 is the electron beam current, η is the fraction of incident electron backscattered from Montecarlo simulations (~ 0.25), $\bar{E}$ is the mean energy of backscattered electrons (~ 0.65 E_0), and E_i is the ionization energy (related to the band gap of the material: $E_i = 2.8\ E_g + M$, where $0 < M < 1$ depending on the material [3]).

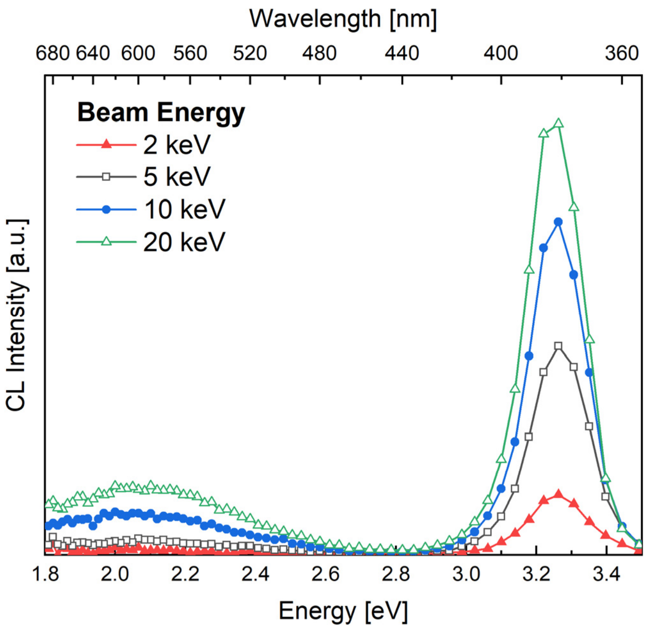

Figure S3. CL spectra of bare ZnO at different electron beam energies.

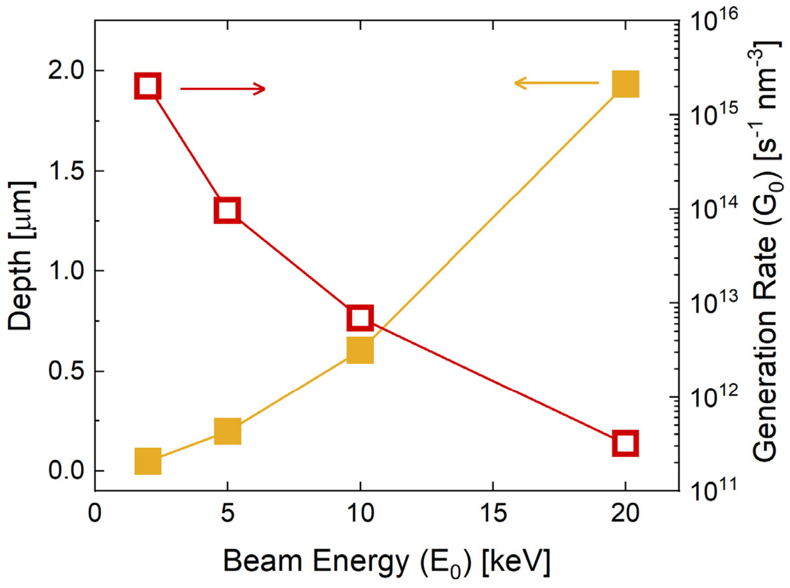

Figure S4. *CASINO* simulation of the probe depth of the electron beam at different beam energies (yellow curve); e-h generation rate (red curve, calculated from Eq.3).

Mott-Schottky Analysis

Using the model of a parallel-plate capacitor, the Mott-Schottky relation can be obtained from [4-7]:

$$\frac{1}{C_{SC}^2} = \frac{2}{\varepsilon_0 \varepsilon_r e N_D}\left(E - E_{FB} - \frac{kT}{e}\right) \qquad (S3)$$

The interface double layer capacitances C_{SC} and C_H (representing the capacitance of the space-charge layer, the capacitance of the Helmholtz double layer at the semiconductor-electrolyte interface) can be treated as two capacitors connected in series. The overall capacitance C is then given by

$$\frac{1}{C} = \frac{1}{C_{SC}} + \frac{1}{C_H} \qquad (S4)$$

It can be assumed that the width of the space-charge layer is much larger than the width of the Helmholtz layer and $C_{SC} \simeq C$.

From the resulting Mott-Schottky plot the flat band potential E_{FB} and the donor density N_D can be now obtained as the intercept with the x-axis and from the slope of the linear part. In particular

$$N_D = \frac{2}{\varepsilon_0 \varepsilon_r e \left(dC_{SC}^{-2}/dE \right)} \qquad (S5)$$

COMSOL simulation

Within a 2D axisymmetric space, a small region of a ZnO NR (50 nm length, 30 nm height) is decorated with an Au nanoparticle (20 nm diameter). The following parameters are used for ZnO: relative dielectric permittivity $\varepsilon_r = 8.3$ [8]; energy gap $E_g = 3.4$ eV; electron (hole) mobility and effective mass $\mu_n = 0.01$ m^{2}V^{-1}s^{-1} ($\mu_p = 0.002$ m^{2}V^{-1}s^{-1}) and $m_{\mathrm{eff,C}} = 0.28\, m_0$ ($m_{\mathrm{eff,V}} = 0.59\, m_0$) [9]; electronic affinity $\chi_0 = 4.1$ eV [10]; donor concentration $N_{D0} = 10^{18}$ cm^{-3}. At room temperature, the computed effective densities of states for electrons and holes are $N_C = 3.6\, x\, 10^{24}$ m^{-3} and $N_V = 1.1 \cdot 10^{25}$ m^{-3}, while the effective Richardson constant for electrons is $A_n^* = 4\pi e m_{\mathrm{eff,\,C}}\, k^2/h^3 = 34$ A K^{-2}cm^{-2} [11]. At the unbiased Schottky contact the Fermi level of Au ($\Phi_{Au} = 4.8$ eV) and that of ZnO are aligned, leading to an energy barrier ($\Phi_B = 0.7$ eV) for electrons [12].

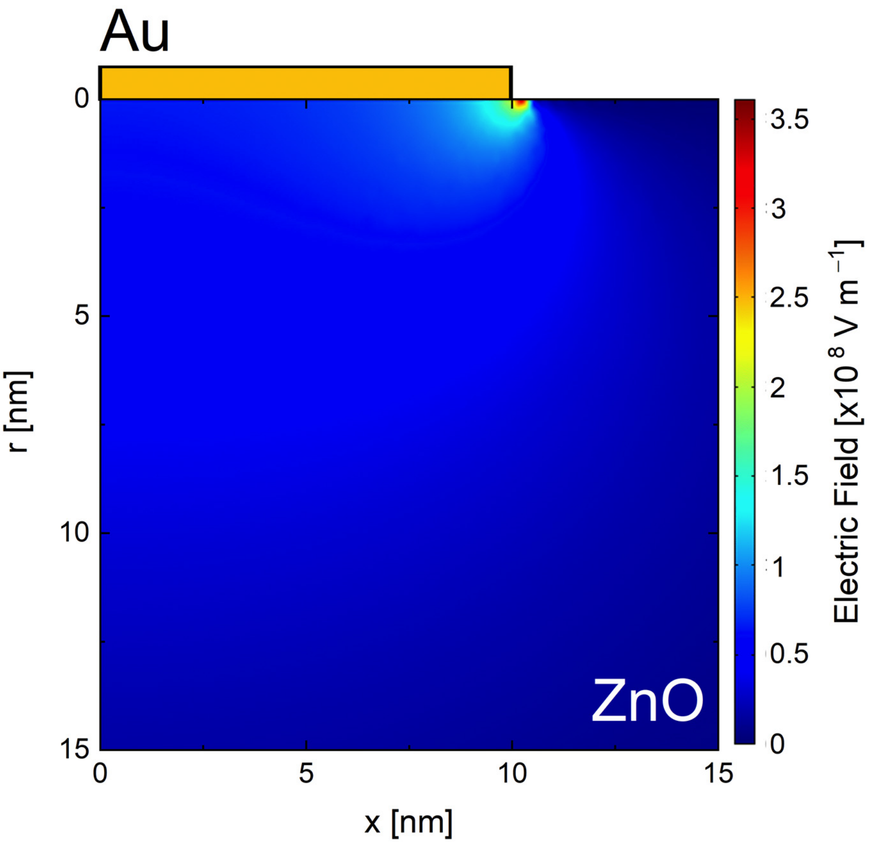

Figure S5. 2D *COMSOL* simulation of the electric field of a single ZnO NR in presence of a Au NP on its surface.

References

1. K. Kanaya, S. Okayama, Penetration and energy-loss theory of electrons in solid targets, J. Phys. D. Appl. Phys. (1972). https://doi.org/10.1088/0022-3727/5/1/308.
2. F.A. Lukiyanov, E.I. Rau, R.A. Sennov, Depth range of primary electrons, electron beam broadening, and spatial resolution in electron-beam studies, Bull. Russ. Acad. Sci. Phys. (2009). https://doi.org/10.3103/S1062873809040029.
3. G. Agostini, C. Lamberti, Characterization of Semiconductor Heterostructures and Nanostructures, 2008. https://doi.org/10.1016/B978-0-444-53099-8.X0001-2.
4. A.W. Bott, Electrochemistry of Electrochemistry of Semiconductors, Curr. Sep. (1998).
5. R. Beranek, (Photo)electrochemical methods for the determination of the band edge positions of TiO 2-based nanomaterials, Adv. Phys. Chem. (2011). https://doi.org/10.1155/2011/786759.
6. F. Fabregat-Santiago, G. Garcia-Belmonte, J. Bisquert, P. Bogdanoff, A. Zaban, Mott-Schottky Analysis of Nanoporous Semiconductor Electrodes in Dielectric State Deposited on SnO[sub 2](F) Conducting Substrates, J. Electrochem. Soc. (2003). https://doi.org/10.1149/1.1568741.
7. A. Hankin, F.E. Bedoya-Lora, J.C. Alexander, A. Regoutz, G.H. Kelsall, Flat band potential determination: Avoiding the pitfalls, J. Mater. Chem. A. (2019). https://doi.org/10.1039/c9ta09569a.
8. Y. Gao, Z.L. Wang, Equilibrium potential of free charge carriers in a bent piezoelectric semiconductive nanowire, Nano Lett. (2009). https://doi.org/10.1021/nl803547f.
9. C.F. Klingshirn, ZnO: Material, physics and applications, ChemPhysChem. (2007). https://doi.org/10.1002/cphc.200700002.
10. C. Jagadish, S. Pearton, Zinc Oxide Bulk, Thin Films and Nanostructures, 2006. https://doi.org/10.1016/B978-0-08-044722-3.X5000-3.
11. S.M. Sze, K.K. Ng, Physics of Semiconductor Devices, 2006. https://doi.org/10.1002/0470068329.
12. M. Henry and H. Fanet, Physique des semiconducteurs et des composants électroniques. Dunod, Malakoff, France, 2019.
